# Circular Letter to the Physicians of Texas, by the Board of Directors P. M. B. A.

**Published:** 1888-12

**Authors:** 


[In addition to the above, we publish the following letter, received after the above was in type.—Ed.]

An Appeal to the Medical Profession of Texas, and Especially to the Members of the P. M. B. A.

*Brother Physicians:*

We earnestly appeal to you in behalf of the P. M. B. A., and ask you to aid our worthy secretary and treasurer in his endeavors to place the Association upon a basis that would be a pride to its members, and a source of pleasure and comfort, as well as substantial aid to the widows and orphans of our deceased brothers.

The physicians of Texas number, as far as we have been able to ascertain, between four and five thousand. It is true, if one half of that number had been members of the Association, and had responded promptly each with one dollar, it would have been a sufficient sum to have been of material benefit to the widows and orphans of our deceased brothers, but it seems impossible so far, to interest the profession in behalf of the Association, except to a very limited extent. We earnestly therefore appeal to the physicians of Texas, and especially to the present members of the P. M. B. A., to come to our aid in this matter, and we feel confident in a short time the Association will more than double its membership, and it will be an institution the pride of every physician in the State, and one of which every doctor will seek to be a member.

Yours fraternally

GEO. CUPPLES,

R. M. SWEARINGEN,

SAML. A. STONE,

J. W. McLAUGLIN,

And other Directors.

